# Mechanisms of Oogenesis-Related Long Non-coding RNAs in Porcine Ovaries Treated With Recombinant Pig Follicle-Stimulating Hormone

**DOI:** 10.3389/fvets.2021.838703

**Published:** 2022-02-24

**Authors:** Haiguang Mao, Lu Chen, Rupo Bao, Shiqiao Weng, Mengting Wang, Ningying Xu, Lili Qi, Jinbo Wang

**Affiliations:** ^1^School of Biological and Chemical Engineering, NingboTech University, Ningbo, China; ^2^Ningbo Sansheng Biological Technology Co., Ltd., Ningbo, China; ^3^College of Animal Science, Zhejiang University, Hangzhou, China

**Keywords:** lncRNA, ovulation, porcine ovary, rpFSH, reproduction

## Abstract

Reproductive efficiency is of significant importance in pork production for it has a great impact on economic success. Ovulation rate is an early component of reproduction efficiency of pigs, and it contributes to the upper limit of litter size. In this study, we used the newly developed recombinant pig follicle stimulating hormone (rpFSH) instead of traditional PMSG to increase ovulation rate of pigs in order to achieve higher litter size, for it was better at stimulating ovulation, and showed more cheaper and greener. However, relatively little is known about the underlying genetic bases and molecular mechanisms. Consequently, an experiment was carried out in ovaries of replacement gilts to screen the key genes and lncRNAs that affect the fecundity of pigs by RNA-seq technology. Twenty gilts were divided into two groups, including 10 rpFSH treatment pigs and 10 control animals. After slaughtering and collecting the phenotypic data, ovaries of five pigs in each group were selected for RNA-seq. Total RNA was extracted to construct the library and then sequence on an Illumina Hiseq 4000 system. A comprehensive analysis of mRNAs and long non-coding RNAs (lncRNAs) from 10 samples was performed with bioinformatics. The phenotypic data showed that rpFSH treatment groups had the higher (*P* < 0.01) ovarian weight and more mature follicles. The RNA-seq results showed that a total of 43,499 mRNAs and 21,703 lncRNAs were identified, including 21,300 novel lncRNAs and 403 known lncRNAs, of which 585 mRNAs and 398 lncRNAs (*P* < 0.05) were significantly differentially expressed (DE) between the two groups of rpFSH treatment group and controlled group. GO and KEGG annotation analysis indicated that the target genes of DE lncRNAs and DE mRNAs were related to prolactin receptor activity, mitophagy by induced vacuole formation, and meiotic spindle. Moreover, we found that NR5A2 (nuclear receptor subfamily 5, group A, member 2), a target gene of lncRNA MSTRG.3902.1, was involved in regulating follicular development, ovulation, and estrogen production. Our study provided a catalog of lncRNAs and mRNAs associated with ovulation of rpFSH treatment, and they deserve further study to deepen the understanding of biological processes in the regulation of ovaries of rpFSH treatment pigs.

## Introduction

Fecundity is of primary interest in pig husbandry for it plays a vital role in the efficiency of production (1). Litter size, such as total number (of piglets) born (TNB), is one of the most important reproductive traits, which is difficult to be improved by traditional selection because of its relatively low heritability (2). In female animals, the mature oocyte quantity and quality are the two main factors affecting fertility (3). Ovulation is the first determinant factor for litter size, and many reports have shown that selection according to ovulation numbers could significantly increase litter size in sows (4, 5). Thus, in current pig production industry, reproductive hormones are widely used to achieve estrous synchronization and maximum reproductive genetic potential by improving the ovulation rate (6).

At present, batch management production of sow is mainly divided into four parts, including synchronization of sexual cycles, synchronization of follicular development, synchronization of ovulation, and synchronous mating (6). Pregnant mare serum gonadotropin (PMSG) is the most widely used in the synchronization of follicular development (7). In this study, we used the newly developed recombinant pig follicle stimulating hormone (rpFSH) instead of traditional PMSG to increase ovulation rate of pigs in order to achieve higher litter size, for it is better at stimulating ovulation, and shows more cheaper and greener. However, little is known about the underlying genetic bases and molecular mechanisms of the role in ovulation by rpFSH.

The ovary of sows, the most important reproductive organ, is responsible for synthesizing and secreting sex hormones, which are necessary for maintaining the hormone levels and the normal reproductive cycles (8). Follicular formation, ovulation, and luteal formation and regression all occur in the ovaries, and these processes take place repeatedly during mammalian reproduction and regulate reproduction (9). Previous reports have shown that long non-coding RNAs (lncRNAs) are involved in ovarian processes and regulate fertility (10–12).

Long non-coding RNAs (lncRNAs) are from regions of the transcriptome with lengths > 200 nucleotides without the capacity of encoding evident proteins (13). Numerous evidences have indicated that the lncRNAs played important roles in the regulation of gene expression by directly recruiting epigenetic complexes or affecting the transcription process (10, 14, 15). To be more specific, lncRNAs could recruit transcription factors to DNA, segregating micro-RNAs (miRNAs) and destabilizing messenger (m) RNA (16). Therefore, the genetic mechanisms of cell differentiation, cell cycle regulation, epigenetics, and dosage compensation are all involved in the protein inhibition by binding of lncRNAs to miRNAs or to proteins or by miRNAs titration (17). LncRNAs have been reported as important regulatory factors in a variety of biological processes including reproduction, but the regulatory mechanism of lncRNAs in biological processes is largely unknown (18). The effects of lncRNAs on animal reproduction traits had been studied in recent years (19–21). The previous studies demonstrated that lncRNAs played an important role in the regulation of pigeon ovulation and sheep fertility (20, 22). Hu et al. (11) identified the ovarian lncRNAs associated with prolificacy of Large White sows during the follicular and luteal phases of the estrous cycle, and found that lncRNAs in ovaries significantly influenced fertility of pigs. Liu et al. (12) identified the lncRNA and mRNA expression profiles for pig ovaries on days 0, 2, and 4 of the follicular periods in Duroc pigs, and found that lncRNA ENSSSCT00000034907 might play an important role in follicular development. Although several researches have focused on the lncRNA expression profile of pig ovarian tissues, none of these studies have interpreted regulatory networks of lncRNAs for regulation of exogenous hormones on follicular formation in sow production.

In the present study, we are the first to perform transcriptome analysis of ovaries in gilts treated with recombinant pig follicle-stimulating hormone by RNA sequencing. The purpose of this study was to reveal the potential role of the lncRNAs in oogenesis treat by rpFSH and further provide a new insight in molecular mechanisms involved in the ovulation and fecundity in pigs. Our data provide a basis to understand the functional role of lncRNAs in improving reproductive rate performance in pigs.

## Materials and Methods

### Animal and Ovary Collection

Twenty young gilts with the same genetic background were divided into two groups (reFSH treatment group and control group) with 10 gilts in each group. All the experiment gilts were born in the same day, raised in the same breeding environment, and fed by the diet according to the Nutrient Requirements of Swine Eleventh Revised Edition 2012. At 210 days of age, altrenogest was administered uniformly for 18 consecutive days, and each pig was fed 20 mg per day. After 18 days, all the pigs were stopped feeding altrenogest for 2 days. The guilts of rpFSH treatment group were injected 1,000 IU per pig, and the control group was injected the same volume of normal saline. The rpFHS (recombinant pig follicle-stimulating hormone) is a new reproductive hormone jointly developed by us and Ningbo Sansheng Biological Technology Co., Ltd. It is a protein-like hormone expressed in CHO. We used the newly developed rpFSH instead of traditional PMSG to increase ovulation rate of pigs in order to achieve higher litter size, for it was better at stimulating ovulation, and showed more cheaper and greener.

The ovary samples of the selected pigs were collected at 234 days of age after removing surface follicles and immediately frozen in liquid nitrogen to isolate RNA. Five samples from each group were randomly selected for sequencing. All animal procedures were approved by the Animal Welfare Committee of Zhejiang University.

### RNA Isolation, Library Preparation, and Sequencing

The total RNA was isolated and purified from ovaries by TRIzol reagent (Invitrogen, Carlsbad, CA, USA) by the manufacturer's instructions. The amount and purity of the RNA samples were quantified by the NanoDrop ND-2000C (Thermo, USA). The extracted RNA integrity was measured by Agilent 2100 with RIN number > 7.0. Approximately 5 μg of the extracted total RNA was used to remove rRNA by the instructions of the Ribo-Zero™ rRNA Removal Kit (Illumina, San Diego, USA). After removing the rRNAs, the rest of RNAs were fragmented into small pieces by divalent cations with high temperature. Afterwards, the cleaved RNA fragments were reversed into cDNA, and it was used to synthesize the U-labeled second-stranded DNAs with RNase H, *E. coli* DNA polymerase I, and dUTP. The average insert size of the final cDNA library was about 300 ± 50 bp. Finally, Illumina Hiseq 4000 (LC Bio, China) was used for the paired-end sequencing according to the recommended protocol of the apparatus. The RNA sequencing data has been uploaded in GEO with the accession number GSE192605.

### Quality Control and Mapping

The low-quality reads, including low quality bases, adaptor contamination, and undetermined bases, were removed by Cutadapt. Sequence quality was then verified by FastQC. Hisat2 (23) and Bowtie2 (24) were used to map the reads to the genome of pigs (*Sus scrofa*) in NCBI (https://ftp.ncbi.nlm.nih.gov/genomes/all/GCF/000/003/025/GCF_000003025.6_Sscrofa11.1/GCF_000003025.6_Sscrofa11.1_genomic.fna.gz). The mapped reads for each sample were then assembled by StringTie. All the transcripts from pig ovaries was combined to reconstruct a comprehensive transcriptome by a Perl script. Ballgown (25) and StringTie (26) were performed to estimate the expression levels of all the transcripts after the final transcriptome were generated.

### Identification of lncRNAs

First of all, transcripts shorter than 200 bp or overlapped with known mRNAs were discarded. CNCI (27) and CPC (28) were applied to predict transcripts with coding potential. All the transcripts of CNCI scores < 0 and CPC score < −1 were removed. The rest transcripts were considered as lncRNAs.

### Different Expressed mRNAs and lncRNAs Analysis

The mRNA and lncRNA expression levels were calculated with FPKM by StringTie. The DE mRNAs and DE lncRNAs were selected with the statistical significance (*P* < 0.05) and with log2 (fold change) < −1 or log2 (fold change) > 1 by R package-Ballgown.

### Prediction of Target Gene and Functional Analysis of lncRNAs

The cis-target genes of lncRNAs were predicted to explore the function of lncRNAs, which were likely play a cis role on the neighboring target genes. In this study, the coding genes in 100,000 downstream and upstream were selected using python script (29). Afterwards, functional analysis of the target genes of lncRNAs was performed by the BLAST2GO (30).

### GO and KEGG Enrichment Analysis

Gene Ontology (GO) terms and Kyoto Encyclopedia of Genes and Genomes (KEGG) pathway enrichment analysis were performed to explore the biological processes, which might contribute to further understanding the biological functions of DE lncRNAs in pigs treated with rpFSH.

### RNA-seq Result Validation by qRT-PCR

Six lncRNAs (MSTRG.16871.1, MSTRG.29090.1, MSTRG.34178.2, MSTRG.30735.1, MSTRG.33729.1, and MSTRG.33864.1) and six mRNAs (BET1L, RSAD2, CCR1, DRC7, LRRC46, and CFAP161) representing differential expression levels of RNA-seq from the 10 pig ovaries were randomly selected to perform qRT-PCR. The qRT-PCR was performed by the ABI Step One Plus system (Applied Biosystem, Carlsbad, CA, USA) with SYBR Premix Ex Taq kit (TaKaRa, Dalian, China), and the primers were shown in Supplementary Table S1. Relative gene expression levels were quantified and normalized by β*-actin* gene using 2^−ΔΔCt^ method with three independent biological replicates. All the measurements were performed in triplicate. Bonferroni correction method was used in the multiple comparison.

## Results

### Phenotypic Data Analysis

**Figure 1:**
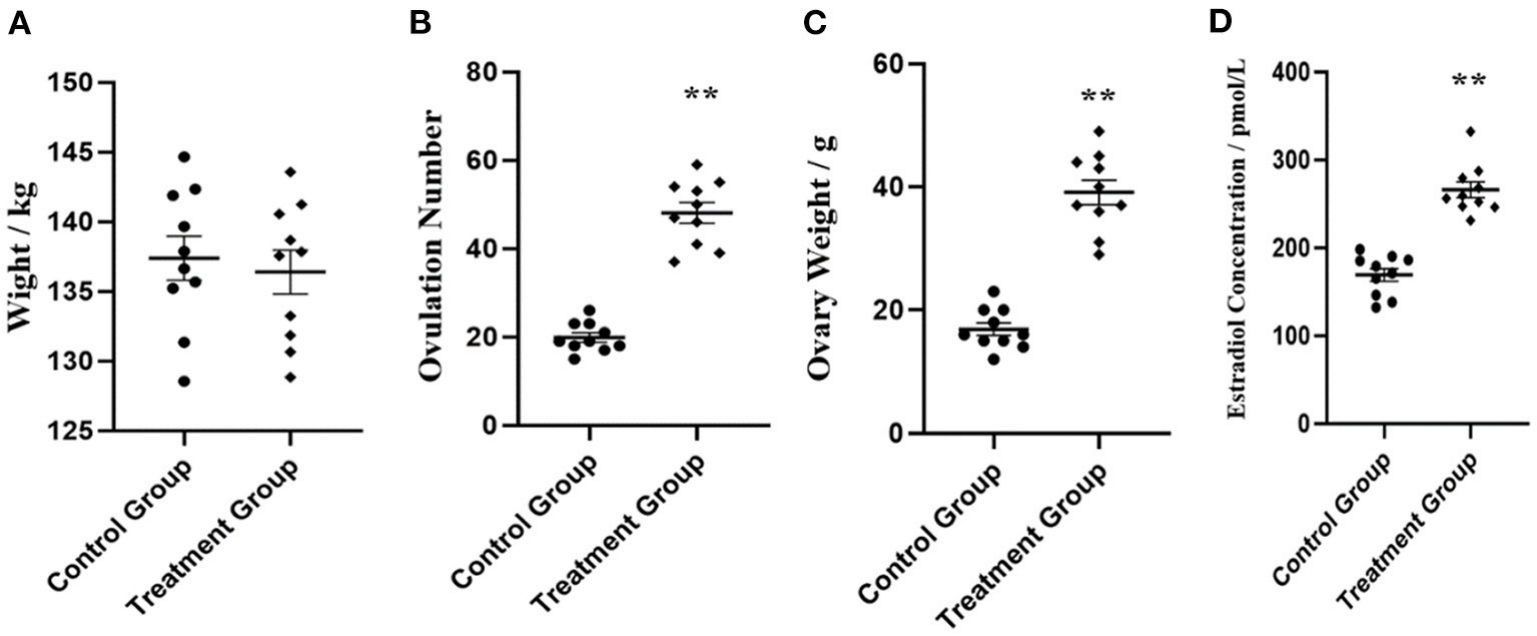
Phenotypic data analysis of experiment gilts between control group and rpFSH treatment group (*n* = 10). **(A)** Live weight analysis. **(B)** Ovulation number analysis of bilateral ovaries. **(C)** Ovary weight analysis of bilateral ovaries. **(D)** Estradiol concentration analysis in serum. **P < 0.01.

Four phenotypic traits were collected, including live weight, ovulation number bilateral ovaries, ovary weight of bilateral ovaries, and estradiol concentration in serum (*n* = 10). As shown in Figure 1, no significant (*P* > 0.05) body weight was shown between the control group and rpFSH treatment group. As expected, the rpFSH treatment group showed significantly higher (*P* < 0.01) ovulation number of bilateral ovaries, ovary weight of bilateral ovaries, and estradiol concentration in serum.

### Sequencing Date Summary

A total of 132.99 Gb raw date was obtained from ten libraries. In detail, 84853980, 87895596, 81995902, 93361996, and 96713942 raw reads were generated from the control group (Control1, Control2, Control3, Control4 and Control5); 88923972, 93496552, 83448796, 86664168, and 89271778 raw reads were generated from the treatment group (Treat1, Treat2, Treat3, Treat4, and Treat5). All the raw reads were filtered to obtain the clean reads, which were mapped to the Cliv_1.0 version of the pig (*Sus scrofa*) genome sequence, with the mapping ratio ranging from 91.13 to 93.07%. The detailed dates are shown in Supplementary Table S2.

### Identification of lncRNAs and mRNAs in Pig Ovaries

As shown in Supplementary Table S3, a total of 21,703 putative lncRNAs were identified from the 10 libraries, including 21,300 novel lncRNAs and 403 known lncRNAs. Regarding the genomic locations of the lncRNAs, 11,918 were intronic (54.91%), 605 were bidirectional (2.79%), 1,683 were sense (7.75%), 6,082 were intergenic (28.03%), and 1,415 were antisense lncRNAs (6.52%).

**Figure 2:**
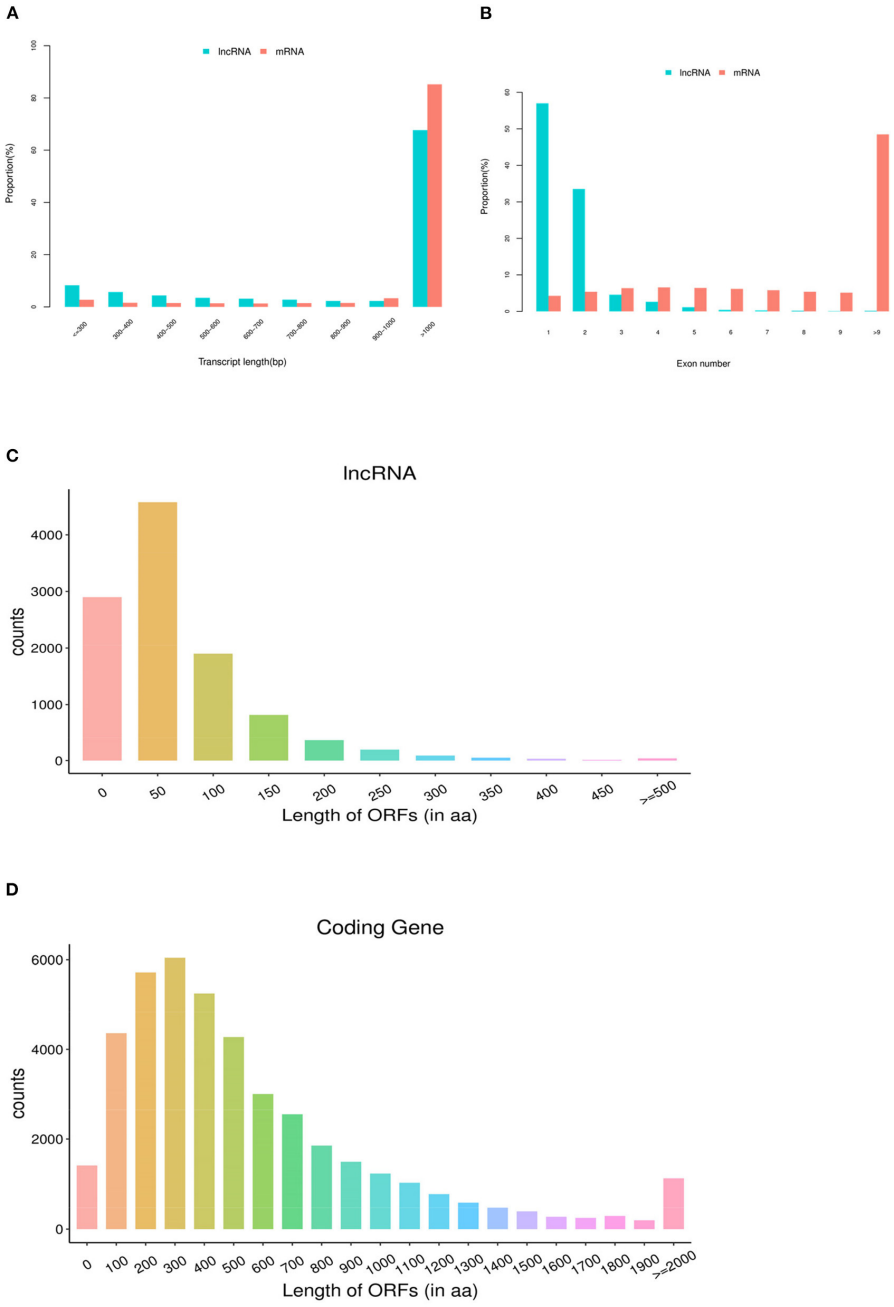
Genomic features of lncRNAs in pig ovaries. **(A)** The transcript length distribution of lncRNAs and mRNAs. **(B)** The exon number distribution of lncRNAs and mRNAs. **(C)** The ORF length distribution of lncRNAs. **(D)** The ORF length distribution of mRNAs.

In this study, the average length of identified lncRNA transcripts is 2,411 bp, which shows shorter than 4,896 bp length of the mRNA transcripts (Figure 2A). Moreover, the number of exons of lncRNAs is 1.63 on average, which is less than that of mRNAs (11.81 on average).

As shown in Figure 2B, 95.09% of lncRNAs contain three or fewer exons, while 77.42% of mRNAs contain five or more exons. In addition, lncRNAs found in the present study showed shorter open reading frames (ORFs) than mRNAs of ovarian tissues in pigs (Figures 2C,D).

### Identification of DE mRNAs and DE lncRNAs

**Figure 3:**
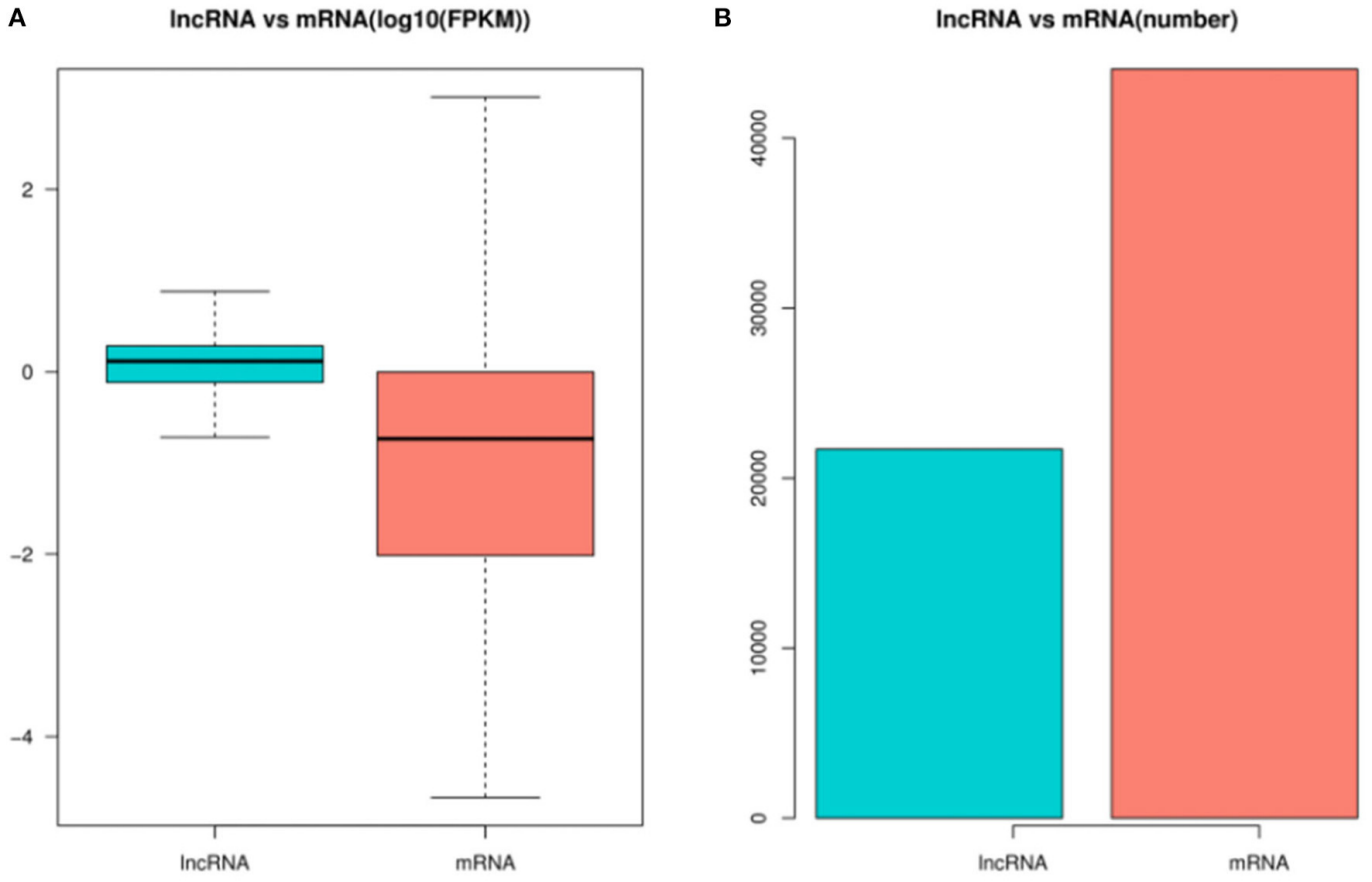
The expression levels and amounts of lncRNAs and mRNAs. **(A)** Boxplots of lncRNA and mRNA expression levels (with log10 FPKM method) in the control group and treatment group. **(B)** The numbers of lncRNAs and mRNAs in pig ovaries in in the control group and treatment group.

In order to identify the mRNAs and DE lncRNAs between the control group and treatment group, we calculated the DE mRNAs and DE lncRNA expression levels with FPKM levels in pig ovaries. Figure 3A shows that the lncRNA expression levels were higher than mRNA expression levels in this study, while Figure 3B shows that the number of lncRNAs was less than that of mRNAs.

**Figure 4:**
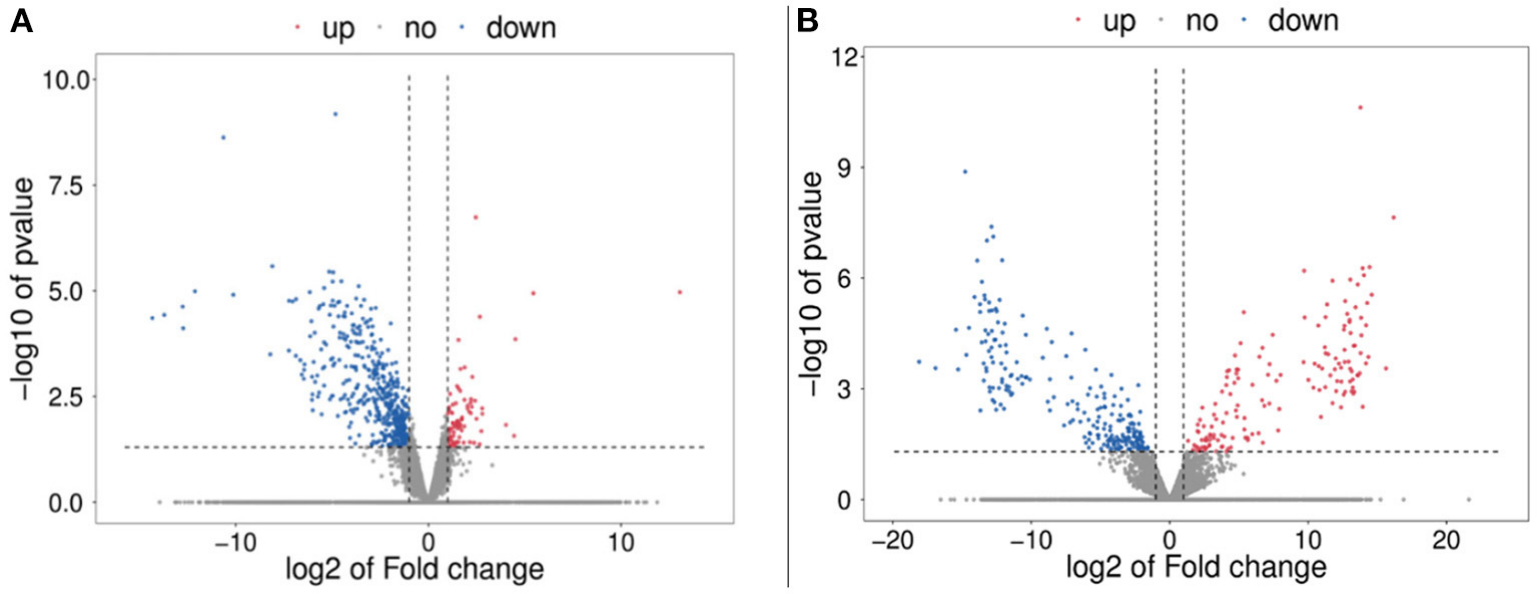
Volcano plot of the differential expression of mRNAs and lncRNAs in pig ovaries between control group and treatment group. **(A)** Differential expression of mRNAs. The blue points denote significantly downregulated mRNAs, while the red points denote significantly upregulated mRNAs. **(B)** Differential expression of lncRNAs. The blue points denote significantly down-regulated lncRNAs, while the red points denote significantly upregulated lncRNAs.

A total of 585 DE mRNAs (Supplementary Table S4) and 398 DE lncRNAs (Supplementary Table S5) were identified between the control group and treatment group. Compared with the control group, 85 mRNAs and 155 lncRNAs were significantly upregulated, while 500 mRNAs and 243 lncRNAs were downregulated. The volcano plot of the DE mRNAs and DE lncRNAs was shown in Figure 4.

### Functional Enrichment of DE mRNAs

**Figure 5:**
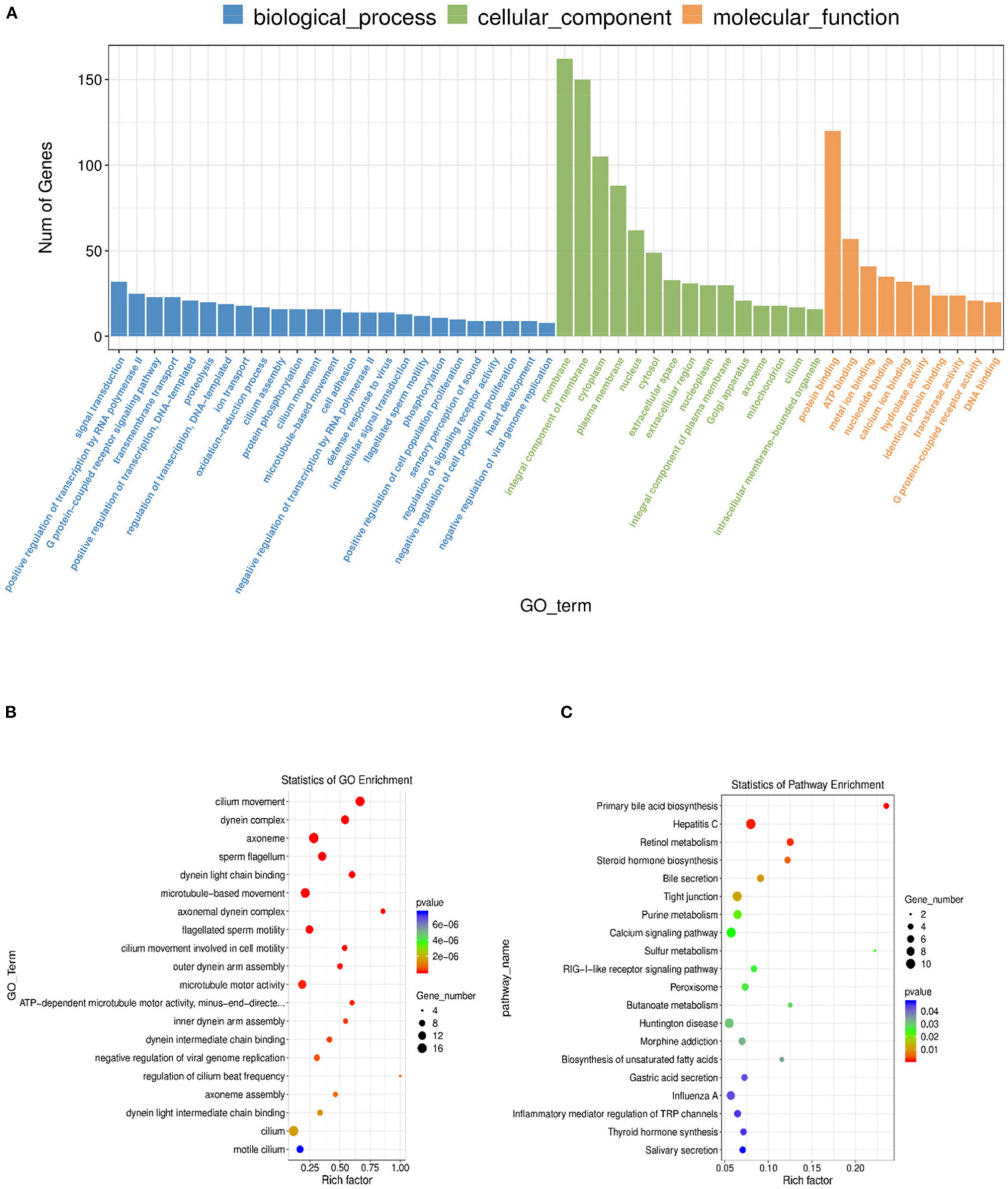
GO and KEGG analysis of differential mRNA expression. **(A)** Histogram of GO enrichment of DE mRNAs. **(B)** Scatter plot of GO enrichment for DE mRNAs. **(C)** Scatter plot of KEGG enrichment for DE mRNAs.

Gene Ontology (GO) was performed to analyze the main functions of the obtained DE mRNAs. A total of 2,417 GO terms with functional annotation information were enriched for 585 DE mRNAs. As shown in Supplementary Table S6, 354 GO terms significantly (*P* < 0.05) enriched in the GO analysis results of DE mRNAs. As shown in Figures 5A–C, the significantly enriched GO terms of DE mRNAs involved cilium movement, dynein complex, axoneme, sperm flagellum, and dynein light chain binding. KEGG pathway analysis showed 20 significantly (*P* < 0.05) enriched pathways, such as primary bile acid biosynthesis, hepatitis C, retinol metabolism, steroid hormone biosynthesis, and bile secretion. The detailed information was shown in Supplementary Table S7.

### Cis-Regulatory Roles of DE lncRNAs in Ovarian Tissues of Pigs

**Figure 6:**
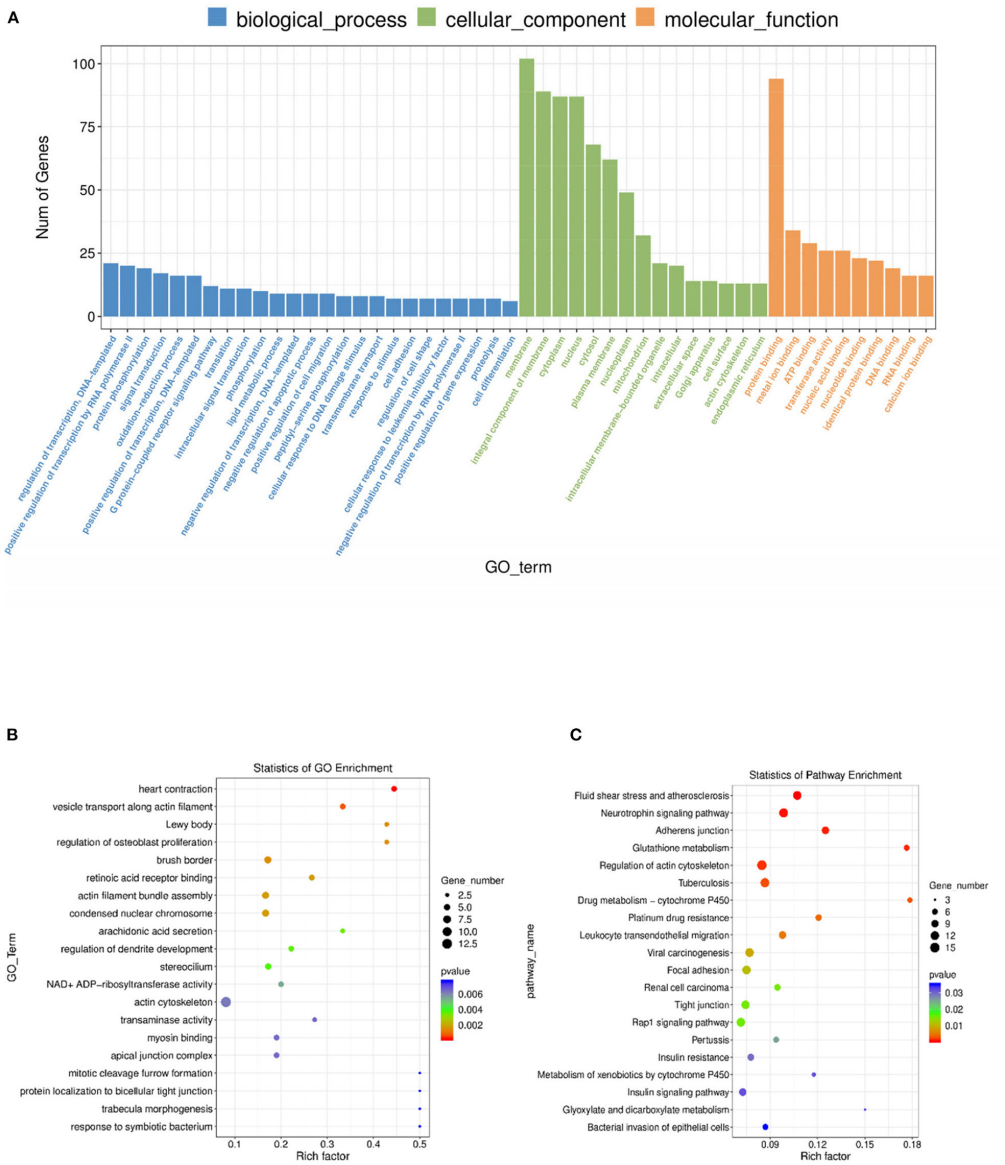
GO and KEGG analysis of differentially lncRNA expression. **(A)** Histogram of GO enrichment of DE lncRNAs. **(B)** Scatter plot of GO enrichment for DE lncRNAs. **(C)** Scatter plot of KEGG enrichment for DE lncRNAs.

To further investigate the regulatory functions of the lncRNAs in the ovarian tissues of pigs, we forecasted the cis-regulated target genes of the differently expressed lncRNAs between the control group and treatment group. In this study, 62 potential lncRNA target genes were found, with 100 kbp as the cutoff (Supplementary Table S8). As shown in Supplementary Table S9, GO analysis revealed 220 significant (*P* < 0.05) GO terms based on the cis-regulated target genes. The differentially expressed lncRNA target genes were founded to be related with biological process including heart contraction, vesicle transport along actin filament, regulation of osteoblast proliferation, and actin filament bundle assembly. The main molecular function and cellular component categories were related to the lewy body, brush border, retinoic acid receptor binding, and condensed nuclear chromosome (Figures 6A,B). The KEGG analysis of DE lncRNAs revealed that the target genes of those lncRNAs were mainly enriched in fluid shear stress and atherosclerosis, neurotrophin signaling pathway, adherens junction, glutathione metabolism, and regulation of actin cytoskeleton (Figure 6C, Supplementary Table S10). Based on the prediction of DE lncRNA-gene pairs in cis-regulation, the first 5 and the last 5 lncRNA-gene pairs were listed in Table 1 by the Pearson correlation coefficient, and the regulation directions of the first 5 lncRNA-gene pairs showed the same, while the last 4 pairs were opposite.

**Table 1:** Differentially expressed lncRNA-gene pairs between high and low egg production groups.

**Gene name**	**lncRNA transcript name**	**Cislocation(bp)**	**Pearson correlation coefficient**
DOCK4	MSTRG.16790.2	100K	1
WDR49	MSTRG.8936.1	100K	1
ENSSSCG00000033862	MSTRG.23824.1	100K	1
SCP2	MSTRG.29550.1	100K	1
ENSSSCG00000033862	MSTRG.23822.2	100K	1
PXYLP1	MSTRG.8684.1	100K	−0.21
PXYLP1	MSTRG.8685.1	100K	−0.20
PHF21A	MSTRG.17683.2	100K	−0.20
PHF21A	MSTRG.17683.2	100K	−0.11

### Co-enriched GO Terms of DE lncRNAs and mRNAs

In order to investigate the crucial pathways of rpFSH to gilt ovaries, a total of five significantly enriched GO terms were identified in both DE lncRNA target gene enrichment and DE mRNA enrichment (Table 2). The significantly co-enriched GO terms were involved in the prolactin receptor activity, brush border, protein ADP-ribosylation, mitophagy by induced vacuole formation, and meiotic spindle, of which one pathway was involved in molecular function, and two pathways were involved in cellular component and biological process, respectively.

**Table 2:** Co-enriched GO terms of DE lncRNA and DE mRNA.

**GO term**	**GO function**	***P*-value**
Prolactin receptor activity	molecular_function	0.04
Brush border	cellular_component	0.00
Protein ADP-ribosylation	biological_process	0.02
Mitophagy by induced vacuole formation	biological_process	0.04
Meiotic spindle	cellular_component	0.02

### DE lncRNAs and DE mRNA Validation by qRT-PCR

**Figure 7:**
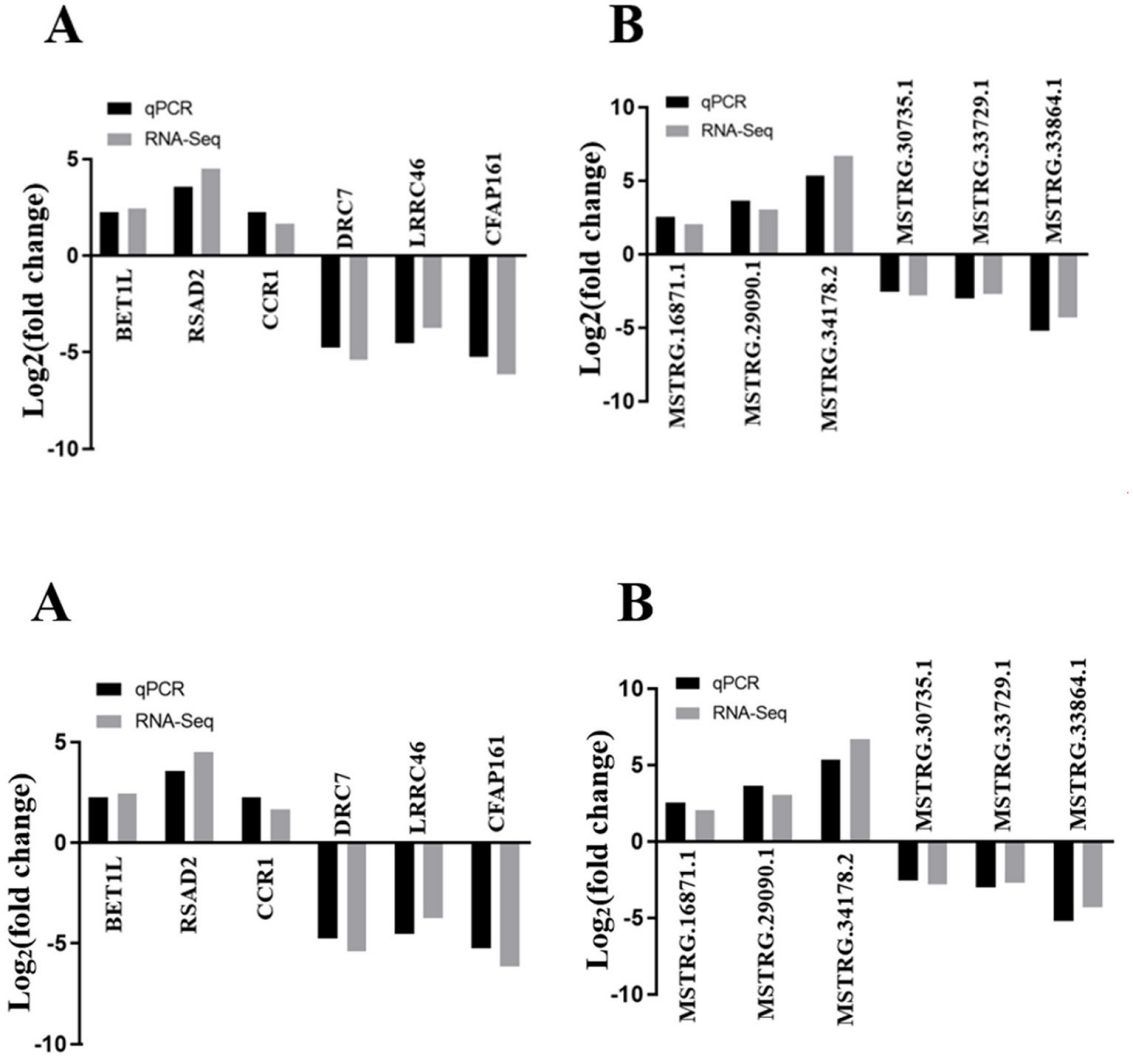
The validation of RNA-seq by qRT-PCR (*n* = 5). **(A)** qRT-PCR validation of six mRNAs. **(B)** qRT-PCR validation of six lncRNAs.

**Figure 8:**
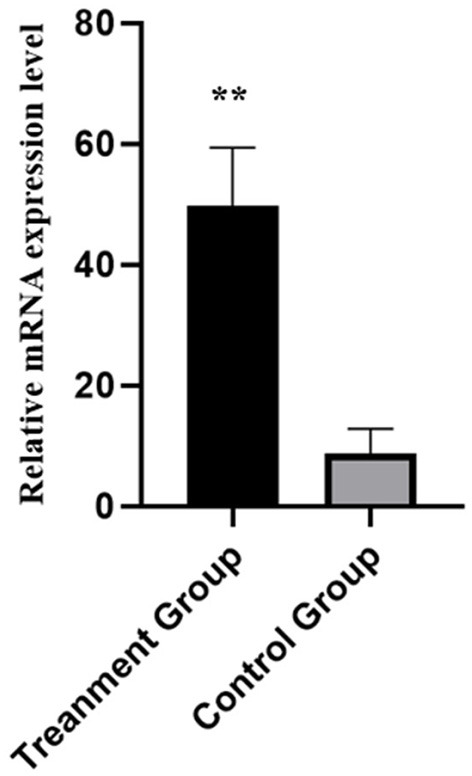
Relative mRNA expression levels of pig *NR5A2* gene in ovaries between rpFSH treatment group and the control group (*n* = 5). **indicate significant differences (*P* < 0.01).

Six DE lncRNAs and six DE mRNAs were selected at random to validate the RNA-seq result by qRT-PCR. The relative fold changes of expression levels performed by qRT-PCR were consistent with the results of RNA-seq data (Figure 7), suggesting that the identification of transcripts and estimation of abundance were highly credible in this study. What is more, the relative mRNA expression level of NR5A2 in the rpFSH group was significantly higher (*P* < 0.01) than that in the control group (Figure 8).

## Discussion

The oogenesis and ovulation in mammals are relatively complex biological processes, which are well-coordinated and regulated by coding and non-coding RNAs (12). Ovulation number is a very crucial trait in pigs for it determines the maximum litter size (4, 5), which is one of the most important economic traits and is difficult to be improved by traditional selection because of its relatively low heritability (2). To achieve the aim of high ovulation rate in pigs, scientists had made great effort on the regulation mechanisms of animal reproduction, which had been greatly driven to perform related gene screening involved in the reproductive regulation of pigs and to mediate the process for increasing the litter size (1, 3, 9, 31). In the last few decades, the whole genomes of pigs had been continuously published, which contributed to facilitate the studies on the transcriptome in pigs (9, 10). In this study, gilts treated with rpFSH showed higher ovulation number and estrogen concentration, which might contribute to improving the litter size of pigs. Many researches have been reported that lncRNAs could regulated reproduction of pigs (10–12), but none of these studies have interpreted regulatory networks of lncRNAs for regulation of exogenous hormones on follicular formation in sow production. Therefore, in this study, we performed transcriptome analysis of ovaries in pigs treated with rpFSH by RNA sequencing. Finally, we identified 398 DE lncRNAs and 585 DE mRNAs in ovaries between the rpFSH treatment group and control group.

In the last decade, a number of researches had reported that lncRNAs played important roles in the oogenesis and ovulation in ovaries among different species, including pigs, cattle, mice, and sheep (11, 21, 32, 33). The present study is the first to report the transcriptome profiling of lncRNAs and mRNA in pig ovaries treated with rpFSH. Our sequencing results showed that the lncRNAs identified in the present study had shorter transcript lengths and fewer exons; this was consistent with the previous research results (9, 20), implying that the lncRNA sequencing result of this study was credible. The RNA-seq results showed that 32.32% of identified lncRNAs were shorter than 1,000 bp, while only 19.18% of identified mRNAs were shorter than 1,000 bp. In addition, the average expression levels of lncRNAs in this study were much higher (*P* < 0.05) than those of mRNAs in ovaries of pigs, suggesting that the lncRNAs in pig ovaries might play important roles in oogenesis and ovulation.

Many studies have found that numerous signaling pathways and regulatory mechanisms are taken part in the regulation of oogenesis and ovulation in animals (11, 21, 32, 33). In the current study, we performed GO terms and KEGG pathways analysis to further identify the biological functions of the target genes of DE mRNAs and DE lncRNAs related to oogenesis and ovulation in pig ovaries. The results revealed that both of these DE mRNAs and DE lncRNAs were participated in the regulation of protein binding, ATP binding, cell differentiation, and transcription by RNA polymerase II. It can be illustrated that ATP binding has been reported to participate in the oogenesis regulation (34). The RNA polymerase II was reported to be involved in the combinatorial control of Spo11 splicing, which is timely regulated during meiosis (35).

The previous studies have shown that the expression of lncRNAs can regulate the expression of the neighboring mRNAs by transcriptional repression or coactivation patterns and had high correlations with the expression levels with the adjacent genes (36, 37). In consequence, we speculated that there was a genetic mechanism that the lncRNAs could significantly affect the oogenesis and ovulation by mediating the putative regulation of the corresponding target mRNAs in pig ovaries. In the current study, the DE cis-target genes, which were located within 100 kb downstream and upstream of the 398 DE lncRNAs, were selected to predict the potential biological functions in the putative regulation of oogenesis and ovulation in pigs. The result suggested that the DE coding gene NR5A2 (nuclear receptor subfamily 5, group A, member 2) might be regulated by the DE lncRNA MSTRG.3902.1, and NR5A2 was significantly upregulated in rpFSH treatment group.

NR5A2, also known as liver receptor homolog-1 (LRH1), is an important orphan receptor, which belongs to the nuclear receptor subfamily NR5A (38). NR5A2 plays a significant role in somatic cell reprogramming, embryonic development, steroid hormone production, and follicle and oocyte development (39). In adult mammals, NR5A2 is mainly expressed in liver, intestine, and ovary tissues, especially in ovarian tissues where it is highly expressed (40, 41), indicating that NR5A2 might play an important role in the reproduction of female animals. NR5A2 gene knockout mice showed ovulation dysfunction and infertility, suggesting that NR5A2 is necessary for follicular development and ovulation in mammals (42, 43). The expression level of *NR5A2* gene in ovary was positively correlated with estrogen content (40), and *NR5A2* regulated porcine follicular estrogen secretion and granular cell apoptosis by targeting *CYP19A1* and *CYP11A1* genes (42). Moreover, the polymorphism of *NR5A2* gene showed significant association with litter size in Hu sheep (33). Our analysis result showed that the mRNA expression level of *NR5A2* in rpFSH treatment group was significantly higher (*P* < 0.01) than that of the control group; thus, we inferred that the NR5A2 might play an important role in the oogenesis and estrogen secretion. Therefore, we speculated that the *NR5A2* gene could be a candidate gene for further study in terms of how it affected oogenesis and ovulation in rpFSH treated pigs.

In conclusion, this study is the first comprehensive description of mRNA and lncRNA profiles of porcine ovaries treated with rpFSH. Several DE lncRNAs are revealed to be associated with ovulation number treated with rpFSH. Moreover, the DE lncRNAs identified in the present study could provide new insights for further understanding the mechanism of ovulation in pigs. The lncRNA MSTRG.3902.1 might play an important regulatory role in ovulation treated with rpFSH by affecting its potential target gene NR5A2. Therefore, lncRNA MSTRG.3902.1 might be a potential candidate lncRNA for regulating oogenesis in pigs treated with rpFSH, and more detailed studies should be carried out to verify the results.

## Data Availability Statement

The datasets presented in this study can be found in online repositories. The names of the repository/repositories and accession number(s) can be found at: https://www.ncbi.nlm.nih.gov/geo/query/acc.cgi?acc=GSE192605.

## Ethics Statement

The animal study was reviewed and approved by the Animal Welfare Committee of Zhejiang University. Written informed consent was obtained from the owners for the participation of their animals in this study.

## Author Contributions

HM analyzed the data and drafted the manuscript. LC and RB collected the tissue samples and analyzed the data. MW performed the qRT-PCR. SW and NX provided suggestions for this study. LQ and JW conceived the project and designed the experiments. All authors contributed to the article and approved the submitted version.

## Funding

The current work was funded by Ningbo Science and Technology Innovation 2025 Project (NO. 2018B10095), Ningbo Major Science and Technology Project (NO. 2021Z112), and Talent Introduction Fund of NingboTech University (NO. 20211018Z0216).

## Conflict of Interest

LC, RB, and SW were employed by Ningbo Sansheng Biological Technology Co., Ltd. The remaining authors declare that the research was conducted in the absence of any commercial or financial relationships that could be construed as a potential conflict of interest.

## Publisher's Note

All claims expressed in this article are solely those of the authors and do not necessarily represent those of their affiliated organizations, or those of the publisher, the editors and the reviewers. Any product that may be evaluated in this article, or claim that may be made by its manufacturer, is not guaranteed or endorsed by the publisher.
